# A Priori and a Posteriori Dietary Patterns among Pregnant Women in Johannesburg, South Africa: The NuPED Study

**DOI:** 10.3390/nu13020565

**Published:** 2021-02-09

**Authors:** Cornelia Conradie, Jeannine Baumgartner, Linda Malan, Elizabeth A. Symington, Marike Cockeran, Cornelius M. Smuts, Mieke Faber

**Affiliations:** 1 Centre of Excellence for Nutrition (CEN), North-West University, Potchefstroom 2531, South Africa; jeannine.baumgartner@hest.ethz.ch (J.B.); Linda.Malan@nwu.ac.za (L.M.); Marius.Smuts@nwu.ac.za (C.M.S.); Mieke.Faber@mrc.ac.za (M.F.); 2 Laboratory of Human Nutrition, Institute of Food, Nutrition and Health, ETH Zürich, 8092 Zürich, Switzerland; 3 Department of Life and Consumer Sciences, University of South Africa, Johannesburg 0003, South Africa; syminea@unisa.ac.za; 4 Statistical Consultation Services, North-West University, Potchefstroom 2531, South Africa; Marike.Cockeran@nwu.ac.za; 5 Non-Communicable Diseases Research Unit, South African Medical Research Council, Cape Town 7505, South Africa

**Keywords:** dietary patterns, a priori-defined dietary patterns, a posteriori-derived nutrient patterns, nutrition, pregnancy

## Abstract

Dietary pattern analyses allow assessment of the diet as a whole. Limited studies include both a priori and a posteriori dietary pattern analyses. This study aimed to explore the diet of pregnant women in urban South Africa through both a priori and a posteriori dietary pattern analyses and associated maternal and household factors. Dietary data were collected during early pregnancy using a quantified food frequency questionnaire from 250 pregnant women enrolled in the Nutrition During Pregnancy and Early Development (NuPED) cohort. A priori dietary patterns were determined using the Diet Quality Index-International (DQI-I), and a posteriori nutrient patterns using exploratory factor analysis. Based on the DQI-I, the study population followed a borderline low-quality diet. Three a posteriori nutrient patterns were identified: Pattern 1 “plant protein, iron, thiamine, and folic acid”; pattern 2 “animal protein, copper, vitamin A, and vitamin B_12_”; pattern 3 “fatty acids and sodium”. Pattern 1 was associated with higher dietary quality (*p* < 0.001), lower maternal educational level (*p* = 0.03) and socioeconomic status (*p* < 0.001). Pattern 3 was significantly associated with lower dietary quality. The low dietary quality among pregnant women residing in urban South Africa should be addressed to ensure optimal maternal and offspring health outcomes.

## 1. Introduction

Maternal nutrition during pregnancy is building the foundation for offspring growth, development, and health [1]. With fetal growth being greatly determined by nutrient supply from the mother [2,3], poor maternal diet and lifestyle choices can have a negative effect on pregnancy outcomes and the health of the offspring [4]. Literature reports both maternal under- and overnutrition to influence an offspring’s growth and development [5,6,7], with a poor maternal nutritional status during pregnancy affecting growth, neurodevelopment, and pulmonary, cardiometabolic, and immune function [6]. Long-term effects of maternal malnutrition include an increased risk for chronic illness later in life, and a decreased birth weight within the subsequent generation [6,8]. Pregnancy is thus regarded as a unique period in life during which the health and well-being of the next generation can be influenced [9].

Data on dietary intake among pregnant South African women are, however, sparse [10], with only four articles [11,12,13,14] having been published during the past decade. It is therefore yet unknown as to how the maternal diet affects health outcomes among the South African population. Although both Cormick et al. [11] and Krige et al. [13] found pregnant women to consume diets within the acceptable macronutrient distribution ranges but deficient in key micronutrients, they did not evaluate the overall quality of the diet.

Hu et al. [15] propose the use of dietary pattern analyses as a complemental method in assessing dietary intake. With nutrients not consumed in isolation but as part of a diet comprising of different foods, dietary pattern analyses account for the cumulative and interactive components of the diet to reflect the complexity of the human diet [15,16]. Dietary pattern analyses, therefore, provide a more predictive approach towards studying health outcomes [15] and is thus an imperative methodology in studying the role of the diet during pregnancy in maternal- and offspring health. However, it is only recently that dietary pattern analyses are being used to study the maternal diet during pregnancy. Most data available are from high-income countries with Raghavan et al. [17], indicating that the majority of research available is from healthy Caucasian women with access to medical care. Kibret et al. [18] concluded that research in low-income countries is needed to comprehend the impact of restricted resources on maternal dietary intake.

Dietary patterns can be derived either using the a priori or a posteriori method. The a priori method, also referred to as the hypothesis-oriented approach, entails the use of a dietary quality index to determine adherence to a predefined dietary pattern or dietary recommendation [15,19]. Conversely, the a posteriori method is a data-driven approach whereby dietary patterns are derived through statistical modeling techniques from empirical data [15,20]. A posteriori dietary patterns, therefore, provide insight into dietary behaviors as they reflect on actual food or nutrient intake [21]. Although these two methods are regarded to provide complementary information with regard to the overall diet [21], only a few studies [22,23,24] have used both methods to explore the maternal diet during pregnancy. Furthermore, to our knowledge, all studies reporting on a posteriori dietary patterns during pregnancy used foods or food groups as variables to determine patterns. Moskal et al. [20] though advocated for the use of nutrients as variables to determine empirical dietary patterns, as nutrients are universal in consumption, and allow for easier comparison between populations.

Only one study to date reported on maternal dietary patterns among pregnant South African women. Wrottesley et al. [14] found three distinct food patterns followed by their study population, namely, a “western”, “mixed”, and “traditional” food pattern. To address the sparsity of research, the purpose of this study was to explore the maternal diet of pregnant women residing in urban Johannesburg, South Africa, through both a priori and a posteriori dietary pattern analyses. Maternal- and household factors associated with these patterns are also determined.

## 2. Materials and Methods

### 2.1. Study Design and Participants

This study was embedded within the Nutrition During Pregnancy and Early Development (NuPED) cohort, a prospective study conducted in Johannesburg, South Africa. The details of the NuPED study have previously been described [25]. Data collection took place at early pregnancy (<18 weeks’ gestation), mid-pregnancy (±22 weeks), late pregnancy (±36 weeks), birth, as well as postnatally (6 weeks, 6 months, 7.5 months, and 12 months’ postnatal age). The current analysis reports on data collected at baseline (early pregnancy).

In brief, the recruitment of generally healthy urban pregnant women from primary health care clinics in Johannesburg took place between March 2016 and November 2017. Based on the South African socioeconomic profile, it is mainly the poorer proportion of the population that makes use of medical care provided by primary health care facilities [26]. Women were regarded as eligible for inclusion if they were 18 to 39 years old, less than 18 weeks of gestation with a singleton pregnancy, born within South Africa or in a neighboring country (Lesotho, Swaziland, Zimbabwe, Botswana, or Namibia), and able to effectively communicate in the local languages (English, Afrikaans, Sotho, Zulu or Xhosa). Women born in a neighboring country had to have been living in South Africa for at least 12 months. Pregnant women diagnosed with a non-communicable disease (namely, hypertension, hypercholesterolemia, diabetes, and renal disease), infectious disease (namely, tuberculosis and hepatitis), or serious illness (namely, lupus, cancer, and psychosis) were excluded. Women were also excluded if they used illicit drugs (self-reported) and/or smoked (currently or in the past year). As South Africa has a high prevalence of human immunodeficiency virus (HIV) infections (22.7% of women of reproductive age [27]), HIV status was not considered exclusion criteria for enrolment.

Five-hundred and ninety-five pregnant women were invited to take part in the NuPED study of which 313 (53%) women volunteered. A further 63 women were excluded for not meeting the inclusion and exclusion criteria. In total, 250 pregnant women provided informed consent and were enrolled. Participants were followed-up at a tertiary hospital (Rahima Moosa Mother and Child Hospital) where data collection took place alongside routine maternal care. All 250 pregnant women completed data collection at baseline (<18 weeks’ gestation).

### 2.2. Ethical Considerations

All women eligible and willing to partake in the study were provided with an informed consent form during recruitment. Written informed consent was obtained during the first study visit before any data were collected. The study was conducted according to the guidelines set out by the World Medical Association Declaration of Helsinki. Ethical approval was obtained from both the Human Research Ethics Committees of the North-West University, Potchefstroom (NWU-00186-15-A1) and the University of the Witwatersrand, Johannesburg (M 150968). The City of Johannesburg District Research Committee (Gauteng Department of Health) and the clinical manager at Rahima Moosa Mother and Child hospital provided permission for the study to take place in the relevant clinical setting.

### 2.3. Dietary Intake Assessment

Dietary intake was assessed during early pregnancy using an interviewer-administered quantified food-frequency questionnaire (QFFQ). The 140-item QFFQ was validated previously for energy and nutrient intake in an African population in South Africa [28], and its reproducibility was established [29,30]. Trained fieldworkers used standard probing questions [31] to inquire in as much detail as possible about the type/brand, cooking method, frequency, and amount of all foods and drinks consumed over the past four weeks. Household utensils, together with “dish-up and measure”, food packaging material, and two- and three-dimensional food models, were used to quantify the portion sizes reported. Portion sizes were converted to grams by three registered dietitians/nutritionists using the South African Medical Research Council (SAMRC) Food Quantities Manual [32]. All foods and drinks were coded according to the South African Food Composition Database [33]. Daily energy, macro-, and micronutrient intake were calculated by the SAMRC by linking the coded and quantified dietary intake data to the most recent food composition database available at the time.

The daily dietary intake data are reflective of nutrient intake from food, excluding any micronutrient supplementation used. Maize meal and wheat flour are subject to mandatory micronutrient fortification as per South African legislation [34], and these fortified micronutrients (vitamin A, thiamine, riboflavin, niacin, pyridoxine, folic acid, iron, and zinc) were considered in the nutrient analysis. All participants provided complete dietary intake information.

#### 2.3.1. A Priori Defined Dietary Patterns

The a priori dietary patterns were defined using the Diet Quality Index-International (DQI-I) [35]. Validated for international use and cross-national comparison of dietary quality [36,37], the DQI-I is regarded as a valuable tool for exploring diet quality related to nutrition transition as it accounts for both dietary concerns contributing to chronic disease and undernutrition. South Africa is known to be undergoing a rapid nutrition transition alongside urbanization [38], contributing to a growing burden of malnutrition within the country [39]. In addition, being sensitive towards various dietary practices and allowing for the use of country-specific recommendations in the evaluation of the diet, the DQI-I serves as a good set of guidelines according to which dietary quality of populations can be evaluated [35].

The DQI-I describes all dimensions of a high-quality diet, namely variety, adequacy, moderation, and overall balance. Each of these categories contains components (including both nutrient and food-group indicators) according to which the diet is assessed, allowing for users to determine the aspect(s) of the diet which most likely needs improvement [40]. The four categories of the DQI-I amount to an overall score of 100, with a higher score indicating greater adherence to a higher quality diet. According to the criteria given by Kim et al. [35], a score of less than 60 out of a possible 100 is indicative of a poor quality diet.

The daily dietary intake for the study participants, based on the QFFQ, was scored as specified by Kim et al. [35]. Adjustments to the DQI-I criteria were made as follows: (1) serving sizes were determined both according to the guidelines provided by the South African Food-Based Dietary Guidelines [41] and the United States’ Food Guide Pyramid serving size definitions [42]; (2) adequacy of micronutrient intake (iron, calcium, and vitamin C) was based on the Dietary Reference Intakes for pregnant women [43]; (3) the cut-off values for sodium were adjusted as the QFFQ used does not report on discretionary salt intake. According to a paper by Wentzel-Viljoen et al. [44], it is estimated that about 60% of the total salt intake among the South African population is from processed foods, with the remainder (40%) being the discretionary use of salt during food preparation and meals. Based on this observation, the sodium cut-off values were adjusted as follows: ≤1440 mg (6 points), >1440–2040 mg (3 points) and >2040 mg (0 points). The DQI-I scoring criteria, together with how the study participants scored, are indicated in Table 1.

#### 2.3.2. A Posteriori-Derived Nutrient Patterns

Nutrient patterns were determined from 22 nutrients by exploratory factor analysis [45]. Using the dietary intake data obtained from the QFFQ, total protein was divided into animal- and plant protein, and total fat into saturated-, monounsaturated- and polyunsaturated fatty acids. Carbohydrate intake was reported as total carbohydrate- and total sugar intake. The correlation structure of the nutrient intake data was explored for stability using the Spearman and Pearson correlation coefficients. The difference between the coefficients was minimal (less than 0.1) and, therefore, raw nutrient data were used for the analysis. Nutrient data were energy-adjusted according to the nutrient density model proposed by Willet et al. [46]. The principal factor method was applied with the correlation matrix, and the reliability of the factor analysis verified using the Kaiser–Meyer–Olkin measure of sampling adequacy and Bartlett’s test of sphericity. A Kaiser–Meyer–Olkin value of more than 0.5 and significance (*p* < 0.05) on Bartlett’s test were regarded as adequate. Factors were retained and interpreted for further analysis according to the eigenvalues (more than 1.00), scree plot inspection, the proportion of the total variance explained, and the natural interpretability of the factors. Retained factors (patterns) were rotated by Varimax (orthogonal) rotation for a simpler structure and to improve interpretation. Patterns were named and interpreted according to nutrients with an absolute factor loading of equal or greater than 0.40. A positive factor loading indicates a nutrient to be positively associated with the nutrient pattern and a negative loading that a nutrient is negatively associated with the pattern. Factor scores were calculated for each participant to indicate the degree towards which a participant’s nutrient intake conforms to the identified patterns. Factor scores were computed by weighting the standardized intakes of each nutrient with their factor loadings and then summing the nutrients within the respective patterns [47].

### 2.4. Assessment of Maternal and Household Factors

Several maternal and household factors were considered as possible determinants for dietary patterns during pregnancy. Interviewer-administered questionnaires were used to obtain sociodemographic and socioeconomic information, information on household-level food insecurity, medical history, and risk for prenatal depression and fatigue.

Sociodemographic and socioeconomic information obtained included maternal age, ethnicity, country of birth, marital status, educational level, employment status, and household beneficiaries of social support grants. Living standard data were also obtained to allow for classification of the socioeconomic status according to the Living Standard Measure in South Africa [48]. Food insecurity was assessed using the Community Childhood Hunger Identification Project (CCHIP) index [49,50]. This index investigates food insecurity as reflected by access to food on three levels, namely, the household-, adult/individual- and the child level. The extent of household food insecurity (CCHIP index total score) could not be determined as a great proportion of participants (*n* = 113; missing data *n* = 9) were not the parent or caretaker of a child in that household. We therefore only report on the three questions assessing household-level food insecurity, namely: (1) “does your household ever run out of money to buy food”; (2) “do you ever rely on a limited number of foods to feed your family because you are running out of money to buy food for a meal”; (3) “do you ever cut the size of meals or skip meals because there is not enough money for food” [49]. Households were regarded as “at risk for food insecurity” if the participant answered “yes” to one or more of the three questions; “no risk” was considered when the participant answered “no” to all three questions.

Medical history considered in this study included parity, pregnancy symptoms (nausea and/or vomiting experienced), and HIV status (routinely tested as part of antenatal care). Participants recorded pregnancy symptoms (nausea and/or vomiting) during the seven days after enrolment using a pictorial morbidity calendar. Gestational age was confirmed using fetal ultrasonography during early pregnancy. Prenatal fatigue and depression were assessed using the Multidimensional Assessment of Fatigue (MAF) scale [51] and the Edinburgh Postnatal Depression Score (EPDS) [52,53], respectively. MAF measures the severity of fatigue, distress, and the degree to which it influences daily activities. The global fatigue index (GFI) was calculated with higher scores indicating higher levels of fatigue, and a cut-off of 28 out of a possible 50 was used to define women with prenatal fatigue [54]. A cut-off value of ≥9 out of a possible 30 was used for the EPDS to indicate the risk for depressive symptoms [53].

Maternal anthropometric measures were also assessed although not considered as possible determinants for the dietary patterns in this analysis. The exclusion of maternal weight as a determinant is based on the observation by Doyle et al. [55] that the relationship between maternal weight and dietary patterns is more difficult to interpret as maternal weight can serve both as an outcome and a determinant of dietary patterns. Maternal height, weight, and mid-upper arm circumference (MUAC) were assessed during early pregnancy using the standardized methods indicated by the International Society for the Advancement of Kinanthropometry [56]. All measurements were taken twice and recorded to the nearest 0.05 kg for weight and 0.1 cm for height and MUAC. A calibrated scale was used for weight measurements (Seca 813, Hamburg, Germany), a mobile stadiometer for height (Seca 213, Hamburg, Germany), and a non-stretchable metal tape for the MUAC. As weight gain and edema during pregnancy decrease the reliability of the body mass index (BMI) [57], maternal weight at enrolment was adjusted (assuming that 2.5 kg was gained during the first 14 weeks of gestation) according to the gestational weight gain guidelines provided by the Institute of Medicine [58] to calculate an approximate pre-pregnancy BMI. The MUAC cut-offs for underweight and obesity as indicated by the South African Maternity Care guidelines [59] were also considered to evaluate maternal nutritional status, i.e., ≤23 cm considered as underweight and ≥33 cm as obese.

### 2.5. Statistical Analysis

The sample size was set as 250 participants; calculations of the sample size have been previously published [25]. IBM SPSS version 26 (SPSS Inc., Chicago, IL, USA) was used for data processing and statistical analysis. Dietary data were captured in Microsoft Excel, with all other raw data captured in Microsoft Access (Microsoft Corporation, Washington, DC, USA). All dietary data were double-checked to ensure accuracy of food codes used and amounts captured. Twenty percent of all other raw data were randomly checked for correctness.

Data were tested for normality using histograms, q-q plots, and the Shapiro–Wilk test. For normally distributed data, continued variables are reported as mean and standard deviation, and for non-normally distributed data as the median and interquartile range (IQR). Categorical data are reported as frequencies and percentages. Both the DQI-I total scores and nutrient pattern factor scores were divided into tertiles for further analysis. To determine food groups associated with each nutrient pattern, analysis of variance (ANOVA) was used to determine the difference between each food group and the nutrient pattern tertiles. The 140-items of the QFFQ were aggregated into 39 food groups expressed as a percentage of the total energy intake (Tables S1–S3). ANOVA was also used to determine differences between DQI-I scores and the nutrient pattern tertiles. In both instances, post-hoc comparisons for unequal variance were carried out using the Games–Howell test.

Hierarchical multiple linear regression models were applied to test for associations between the dietary patterns and maternal- and household factors. Hierarchical steps were based on the conceptual framework for multiple determinants of diet during pregnancy as proposed by Doyle et al. [55] and were as follows: step 1 investigates all sociodemographic factors (maternal age, level of education, and marital status), step 2 the sociodemographic and socioeconomic factors (employment, social grants received by the household, household-level food insecurity, and living standard measure), step 3 the sociodemographic-, socioeconomic-, pregnancy-related factors (parity, nausea- and/or vomiting experienced) and HIV status, and step 4 the sociodemographic-, socioeconomic-, pregnancy-related factors, HIV status and psychological factors. Significance was defined as *p* < 0.05.

## 3. Results

### 3.1. Characteristics of Study Particpants

Characteristics of the study participants at early pregnancy are shown in Table 2. The median (IQR) age was 27 (24–32) years and the median length of gestation at enrolment 14 (12–16) weeks. Most enrolled women were of black-African descent (88%), followed by women of mixed ancestry (11%). The distribution with regard to the number of previous pregnancies was equal as the same proportion of women (35%) were primiparous and multiparous, respectively. A quarter of the women (26%) had a positive HIV status, and almost half of the women reported experiencing nausea (52%) and vomiting (48%) within seven days after enrolment. Based on the calculated pre-pregnancy BMI, half of the women (52%) entered pregnancy either being overweight or obese. This was confirmed by the MUAC categories which indicated, when compared to the BMI categories, near similar proportions of the study participants to be underweight (4%) and obese (26%). A quarter of the study participants presented with a more severe level of prenatal fatigue (26%) and risk for prenatal depression (26%), respectively.

A quarter of women (23%) reported having tertiary education, with 73% having some form of secondary schooling. Half of the participants (49%) were unemployed at the time of the study, with two-thirds (67%) indicating that no social grants were received by the household. Almost half of all households (47%) were though at risk for food insecurity. Most women (59%) were categorized as have a medium living standard.

### 3.2. Dietary Pattern Analysis

#### 3.2.1. *A Priori* Defined Dietary Patterns

The median total DQI-I score was 60 (54–66) out of a possible 100 (Table 3). The “variety” category had the highest achieved score, followed by the “adequacy” and “moderation” categories. The “overall balance” category was the weakest component of the diet, with a median score of 2 (0–4) out of a possible 10.

About one-third (37%) of participants included at least one serving of each of the five food groups considered for overall food-group variety (meat/poultry/fish/eggs; dairy/beans; grain; fruit and vegetables), with 55% consuming protein from more than three different protein sources per day (Table 1). Vegetable intake was poorer than fruit intake; 63% of the women consumed less than half of the recommended amount of vegetables per day, while 24% consumed less than half of the recommended amount of fruit. A larger percentage of the participants had an intake of more than 50% of the recommendation for grain, fiber, protein, iron, calcium, and vitamin C. Only 11% and 20% achieved the Dietary Reference Intakes for pregnant women for iron and calcium, respectively, from food sources. With regard to the “moderation” category, most participants scored poorly for total fat, saturated fat, and sodium intakes. More than half (59%) of the women had a total fat intake of more than 30% of the total energy intake, with 40% of participants having a daily saturated fat intake of more than 10% of the total energy intake. Almost two-thirds of participants (61%) consumed foods with high sodium content. The high intake of total fat was reflected in the “overall balance” category as a high percentage of participants had a macronutrient (68%) and fatty acid (45%) intake outside of the ratios considered for optimal health.

#### 3.2.2. *A Posteriori*-Derived Nutrient Patterns

Three nutrient patterns were retained by exploratory factor analysis, explaining 57% of the total variance in nutrient intake (eigenvalue > 2.5). Both the Kaiser–Meyer–Olkin measure (0.7) and Bartlett’s test (*p* < 0.001) indicated the analysis to be appropriate for the study sample. Patterns were named according to the dominant nutrients (higher factor loadings) within each factor. The factor loadings and names assigned to each nutrient pattern are indicated in Table 4. The three nutrient patterns were further explored by determining associations between nutrient pattern tertiles and the consumption of food groups based on the percentage of total energy intake.

Pattern 1, named “plant protein, iron, thiamine, and folic acid”, had high positive loadings for plant protein, carbohydrates, fiber, iron, zinc, thiamine, niacin, vitamin B_6_ and folic acid. Animal protein, saturated- and monounsaturated fatty acids, and calcium were negatively associated with this pattern. All micronutrients used for fortification according to South African legislation [34], except for vitamin A and riboflavin, reflected high positive loadings on this factor matrix. Pattern 1 explained 27% of the total variance in nutrient intakes. Based on the associations of this nutrient pattern with the food groups, intake of maize, whole wheat- and brown bread, and legumes were positively associated with nutrient pattern 1 (Table S1) as intake of these food groups was significantly higher across the nutrient pattern tertiles (higher tertile indicating that women are having a dietary intake closer to the respective nutrient pattern (higher factor scores)). Intake of maize increased the most from tertile 1 to tertile 3. Conversely, the intake of cakes, biscuits and pudding, cheese, dairy, fatty foods, fruit juice, processed- and red meat, rice and pasta, salad oils, salad vegetables, sugar-sweetened dairy and -drinks, and sweets and chocolates were significantly lower across the nutrient pattern tertiles. Nutrient pattern 1 is therefore reflective of a “fortified grain and legume” dietary pattern.

Pattern 2, named “animal protein, copper, vitamin A, and vitamin B_12_”, had high positive loadings for animal protein, cholesterol, zinc, copper, vitamin A, riboflavin, and vitamin B_12_, and explained 18% of the total variance. Cheese, chicken, eggs, fish and seafood, nuts and nut spreads, and organ meat and offal were positively associated with nutrient pattern 2 as the intake of these foods was higher across the nutrient pattern tertiles (Table S2). Intake of fatty foods, roast potatoes and chips, sugar, and white bread was, though, significantly lower across the tertiles of nutrient pattern 2. Nutrient pattern 2 is, therefore, reflective of an “animal products” dietary pattern.

Pattern 3, named “fatty acids and sodium”, explained 12% of the variance and had high positive loadings for saturated-, monounsaturated- and polyunsaturated fatty acids, sodium, and vitamin E. Intake of cheese, fatty foods, nuts and nut spreads, processed- and red meat, salad oils, and sugar-sweetened drinks was positively associated with the nutrient pattern 3 (Table S3), with the intake of fatty foods increasing the most across the tertiles. Intake of dairy, fresh and dried fruit, maize, and other cooked porridges, and sugar was significantly lower across pattern 3 tertiles. Nutrient pattern 3 is, therefore, representative of a “fatty- and processed foods” dietary pattern.

#### 3.2.3. Associations between the a Priori and a Posteriori Dietary Patterns

Table 5 indicates the median DQI-I scores for each of the nutrient patterns. One-way ANOVA with post-hoc comparisons indicated the DQI-I total scores (higher scores indicative of higher dietary quality) to significantly increase across the tertiles (tertile 3 presenting higher factor loadings) of nutrient pattern 1 (plant protein, iron, thiamine, and folic acid (fortified grain and legume)), but significantly decrease across nutrient pattern 3 (fatty acids and sodium (fatty- and processed foods)). The category scores “moderation” and “overall balance” were significantly higher across nutrient pattern 1, but significantly lower across the tertiles of nutrient pattern 2 (animal protein, copper, vitamin A, and vitamin B_12_ (animal products)) and nutrient pattern 3. Conversely, the scores for the “variety” category were higher across the tertiles of nutrient pattern 2 (*p* < 0.001) and nutrient pattern 3 (*p* < 0.001), but significantly lower across nutrient pattern 1.

### 3.3. Factors Associated with Dietary Patterns

Table 6 shows the multiple regression coefficients of the dietary patterns retained for the maternal and household factors. When considering the sociodemographic-, socioeconomic-, pregnancy-related factors and HIV status (steps 1 to 3), having a maternal age equal to and older than 27 years (step 3: β = −1.9, SE = 0.9, *p* = 0.046), a lower level of education (<Grade 10) (step 3: β = 2.0, SE = 1.0, *p* = 0.034) and a lower level of living standard (step 3: β = 2.0, SE = 1.0, *p* = 0.049) were significantly associated with higher scores for nutrient pattern 1 (plant protein, iron, thiamine and folic acid (fortified grain and legume)). However, the association with maternal age did not hold when all maternal and household factors were included in the model (step 4), resulting in women of a lower education (β = 2.1, SE = 1.0, *p* = 0.032) and living standard measure (β = 2.0, SE = 1.0, *p* = 0.049) to have higher scores for nutrient pattern 1. For nutrient pattern 3 (fatty acids and sodium (fatty- and processed foods)), only step 1 (sociodemographic factors) had a significant model fit, but none of these factors were significantly associated with nutrient pattern 3 scores.

A maternal age equal to and older than 27 years was also significantly associated with the DQI-I. This association was retained throughout model steps 1 to 3 (step 4 had a non-significant model fit), indicating women equal and older than 27 years of age among our study population to have greater adherence to a diet with a higher dietary quality (step 3: β = −5.8, SE = 1.6, *p* = 0.001). No other maternal and household factors were significantly associated with the dietary patterns among our study population.

## 4. Discussion

We explored the diet of pregnant women living in an urban South African setting during early pregnancy using both a priori and a posteriori dietary pattern analyses. Three distinct dietary patterns were identified, namely, pattern 1 “plant protein, iron, thiamine, and folic acid”; pattern 2 animal protein, copper, vitamin A, and vitamin B_12_”; pattern 3 “fatty acids and sodium”. These nutrient patterns, based on their associations with food groups, represent a “fortified grain and legume”, “animal products”, and “fatty- and processed foods” dietary pattern, respectively. Based on the a priori index, our study population had a borderline low-quality diet (DQI-I total score of 60). Nutrient pattern 1 was positively associated and nutrient pattern 3 negatively associated with dietary quality indicated by the a priori index. Possible determinants for the dietary patterns were found to be maternal age, level of education, and socioeconomic status.

The borderline low-quality diet observed in this study population can be attributed to low scores on the “moderation” and “overall balance” categories of the DQI-I as most participants consumed a diet high in total- and saturated fat, and foods high in sodium. Poor scorings on the total- and saturated fat categories could be due to the use of the stringent cut-off values proposed by Kim et al. [35]. However, these cut-off values are in line with that advocated by the South African Food-Based Dietary Guidelines and were chosen to emphasize the importance of moderate fat intake. A high intake of total fat among pregnant urban South African women was also reported by Krige et al. [13] with two-thirds of their study population consuming more than 30% of their total energy intake from fat. Jordaan [60] indicated similar findings when evaluating the dietary intake among two ethnic groups (including males and females between the ages of 20 and 30 years) of varying socioeconomic backgrounds residing in urban areas. A fat intake of more than 30% of the total energy intake among the urban South African population was indicated by Vorster et al. [38] as an adverse effect of the nutrition transition occurring due to urbanization. A high sodium intake among the South African population is also well described [61,62], hence the implementation of the mandatory legislation in 2016 to reduce the sodium content of certain foodstuffs [63].

In a recent publication, the World Health Organization recommended the intake of total fat to not exceed 30% of total energy as a higher fat intake is often a contributing factor towards unhealthy weight gain in adults [64]. Total fat intake was, however, found to only have a weak positive association with weight gain in the Nurses’ Health Study, with saturated-, trans-, and polyunsaturated fat having stronger positive relationships with bodyweight [65]. Rosqvist et al. [66] found in an overfeeding trial participants to gain similar amounts of weight regardless of consuming a diet high in saturated- or polyunsaturated fat. A diet high in saturated fat though resulted in more visceral adipose fat and liver fat being gained, whereas high intakes of polyunsaturated fat were found to increase lean body tissue [66]. It is, hence, important to consider the quality and type of dietary fat when evaluating health and disease risk [67]. The consensus is that saturated fat should rather be replaced with naturally occurring unsaturated fat, with the intake of industrially produced trans-fat excluded from the diet [64,67]. It should also be considered that often when people follow a diet high in saturated- and trans-fat, other unhealthy lifestyle factors such as a high intake of fast foods and sugar-sweetened beverages, which can contribute to poor health outcomes, are often followed [65].

Nutrient pattern 1 (plant protein, iron, thiamine, and folic acid), which is reflective of a “fortified grain and legume” pattern, represents a more traditional South African diet. The traditional South African diet, which is associated with a lower prevalence of degenerative diseases [68], is characterized by a high fiber and low fat intake as a result of a high plant protein (particularly legumes) and starchy food consumption [29,69]. The representation of nutrient pattern 1 of the traditional diet is supported by a high positive loading for total fiber and high negative loadings for saturated- and monounsaturated fat on the factor matrix, as well as the higher consumption of legumes and lower consumption of products of animal origin and processed foods. Upon determining the associations between the a posteriori-derived nutrient patterns and a priori dietary patterns, nutrient pattern 1 was found to be associated with higher dietary quality. This higher dietary quality is attributed to more favorable scores in the “moderation” and “overall balance” categories of the DQI-I, which is also supported by the loadings of the factor matrix and the lower intake of products of animal origin and processed foods. Dietary variety was negatively associated with this pattern since traditional diets are typically more monotonous [70].

Nutrient pattern 2 (animal protein, copper, vitamin A, and vitamin B_12_) and 3 (fatty acids and sodium) are, conversely, reflective of the nutrition transition. The nutrition transition is described as the increase in the intake of foods of animal origin and processed foods high in fat and sodium as populations adapt to a more modern lifestyle often due to urbanization and economic- and social development [38,71]. These changes in dietary intake are well documented and have been associated with the increasing prevalence of obesity and non-communicable diseases [38]. Although the food groups associated with nutrient pattern 2 were mainly animal products, nutrient pattern 2 was not found to be associated with dietary quality. Nutrient pattern 3, reflecting a “fatty- and processed foods” pattern, was found to be associated with a lower dietary quality, which was mainly driven by poor scorings in the “moderation” and “overall balance” categories. This is supported by high positive loadings on fat (saturated-, monounsaturated- and polyunsaturated fatty acids) and sodium on the factor matrix, together with the higher consumption of fatty foods, processed- and red meat, cheese, nuts and nut spreads, and sugar-sweetened beverages.

Nutrient pattern 1 was found to be adhered to by women with a lower level of education and a poorer socioeconomic status. Considering the positive association between nutrient pattern 1 scores and dietary quality, maternal age (≥27 years) can also be regarded as a determinant for this pattern. In a systematic review on determinants of dietary patterns and dietary quality during pregnancy, Doyle et al. [55] described a social gradient whereby women of older age, a higher education, a higher income, and/or other markers indicating affluence are more likely to follow a healthier dietary pattern [55]. Although our findings were similar with regard to maternal age, a higher quality diet was followed by less affluent women among our study population. This higher dietary quality can be explained by the higher intake of whole wheat- and brown bread, legumes, and maize, which are generally more affordable when compared to fatty foods and processed animal products. Bourne et al. [68] found that individuals within nutrition transition who were more traditionally orientated to frequently be under-employed and therefore have time available to prepare the lower costing maize meal and legume-based dishes, which have relatively long cooking times. Equally, employed city dwellers tend to more frequently choose unhealthy fast foods and convenience foods, which are generally refined and higher in fat due to long commuting times to and from work [68]. Nevertheless, it is important to note that the finding by Doyle et al. [55], in which women of a higher social gradient followed a diet of higher quality, was based on data mainly from high-income countries. This disparity in findings with regard to determinants for dietary patterns between high-income and middle-income countries was also reported by López-Olmedo et al. [72] who indicated individuals from a lower socioeconomic status in Mexico to follow a higher quality diet than individuals from higher socioeconomic status.

In a recently published review, Chia et al. [73] grouped both a priori and a posteriori dietary patterns to identify “healthy” and “unhealthy” patterns to conduct a meta-analysis on the associations between maternal dietary patterns during pregnancy and birth outcomes. A healthier dietary pattern was reported to be associated with a lower risk for preterm birth, with an unhealthy dietary pattern associated with a higher risk for preterm birth and low-birth weight. Considering the associations between the nutrient patterns and dietary quality identified by our study, nutrient pattern 1 reflects a more “healthy” dietary pattern, with nutrient pattern 3 reflecting an “unhealthy” dietary pattern. Based on the findings by Chia et al. [73], it is likely that women with higher adherence to nutrient pattern 3 have an increased risk for adverse pregnancy outcomes. A possible rationale for this is that nutrient pattern 3, which is reflective of a “fatty and processed food” pattern and nutrition transition, increases the risk for overweight, obesity, and non-communicable disease [38]. In one of the only studies to evaluate the change in dietary patterns from before pregnancy and during pregnancy, Crozier et al. [74] found that, although there was a slight difference in the food products consumed, the dietary patterns remain mainly unchanged from before pregnancy. Women following an unhealthy dietary pattern during pregnancy are, therefore, likely to have followed an unhealthy pattern before conception too. Overweight, obesity, and non-communicable diseases both before and during pregnancy are well described to increase the risk for morbidity (gestational diabetes, preeclampsia, and macrosomia) and mortality during pregnancy [7,75,76,77].

A limitation to our study is that the dietary data used to score the a priori dietary patterns were obtained using a QFFQ. As the QFFQ reports on a range of foods or food groups consumed over a long period [78], it is likely that when converting the dietary data to intake per day, a greater variety of foods was reported, which could have led to an overestimation of the “variety” and “adequacy” scorings of the DQI-I. In addition, the QFFQ is not always accurate with regard to the estimated portion sizes consumed as this questionnaire relies heavily on memory [78]. However, the scoring of the DQI-I was performed stringently according to the guidelines given by Kim et al. [35], and we are confident that the results represent an accurate overview of the maternal dietary quality. Furthermore, the associations investigated between the maternal and household factors and the dietary patterns were likely to have been influenced by the relatively small sample size (multiple testing correction was not applied) of our study, as well as our study population being mainly from an urban setting.

## 5. Conclusions

We present a comprehensive analysis of the diet of pregnant women residing in an urban South African setting by including both a priori and a posteriori dietary pattern analyses. Although three distinct a posteriori dietary patterns were followed by participants in our study population, the overall dietary quality was poor. Considering possible poor maternal outcomes associated with lower dietary quality, the poor dietary quality among pregnant South African women, especially women of younger age, should be addressed through nutritional education. It is also advised that further research be carried out to truly understand the impact of the maternal diet on health outcomes among the South African population. Our study also confirms that the a priori and a posteriori methods, based on their methodological construction, evaluate the diet by different means and, therefore, provide complementary and useful information in exploring the diet as a whole. Determining associations between the a priori and a posteriori dietary patterns is also worthwhile as this aids in the better understanding of the a posteriori patterns that are representative of the actual dietary intake.

## Figures and Tables

**Table 1:** Participant scores according to the Diet Quality Index-International (DQI-I) scoring criteria (*n* = 250) [35].

Category and Component	Scoring Criteria		*n* (%) ^1^
**Variety 0–20 points**			
Overall food group variety (meat/poultry/fish/eggs; dairy beans; grain; fruit; vegetables)	0–15 points	>1 serving from each food group/d = 15	92 (37)
		Any 1 food group missing/d = 12	97 (39)
		Any 2 food groups missing/d = 9	38 (15)
		Any 3 food groups missing/d = 6	21 (8)
		>4 food groups missing/d = 3	2 (1)
		None from any food groups = 0	0
Within-group variety for protein source (meat; poultry; fish; dairy/beans; eggs)	0–5 points	>3 different sources/d = 5	137 (55)
		2 different sources/d = 3	72 (29)
		From 1 source/d = 1	31 (12)
		None = 0	10 (4)
**Adequacy 0–40 points**			
Vegetable group ^2,3^	0–5 points	>3–5 servings/d = 5, 0 servings/d = 0	
		>100%	14 (6)
		50–100%	78 (31)
		<50%	158 (63)
Fruit group ^2,3^	0–5 points	>2–4 servings/d = 5, 0 servings/d = 0	
		>100%	93 (37)
		50–100%	97 (39)
		<50%	60 (24)
Grain group ^2,3^	0–5 points	>6–11 servings/d = 5, 0 servings/d = 0	
		>100%	71 (28)
		50–100%	153 (61)
		<50%	26 (10)
Fiber ^2,3^	0–5 points	>20–30 g/d = 5, 0 g/d = 0	
		>100%	149 (60)
		50–100%	97 (39)
		<50%	4 (2)
Protein	0–5 points	>10% of energy/d = 5, 0% of energy/d = 0	
		>100%	241 (96)
		50–100%	9 (4)
		<50%	0
Iron	0–5 points	>100% RDA/d = 5, 0% RDA/d = 0	
		>100%	28 (11)
		50–100%	162 (65)
		<50%	60 (24)
Calcium	0–5 points	>100% AI/d = 5, 0% AI/d = 0	
		>100%	50 (20)
		50–100%	125 (50)
		<50%	75 (30)
Vitamin C	0–5 points	>100% RDA/d = 5, 0% RDA/d = 0	
		>100%	162 (65)
		50–100%	67 (27)
		<50%	21 (8)
**Moderation 0–30 points**			
Total fat	0–6 points	<20% of total energy/d = 6	15 (6)
		>20–30% of total energy/d = 3	88 (35)
		>30% of total energy/d = 0	147 (59)
Saturated fat	0–6 points	<7% of total energy/d = 6	39 (16)
		>7–10% of total energy/d = 3	111 (44)
		>10% of total energy/d = 0	100 (40)
Cholesterol	0–6 points	<300 mg/d = 6	102 (41)
		>300–400 mg/d = 3	46 (18)
		>400 mg/d = 0	102 (41)
Sodium ^4^	0–6 points	<2400 mg/d = 6	44 (18)
		>2400–3400 mg/d = 3	53 (21)
		>3400 mg/d = 0	153 (61)
Empty calories	0–6 points	<3% of total energy/d = 6	47 (19)
		>3–10% of total energy/d = 3	131 (52)
		>10% of total energy/d = 0	72 (29)
**Overall balance 0–10 points**			
Macronutrient ratio ^5^ (carbohydrate:protein:fat)	0–6 points	55~65:10~15:15~25 = 6	10 (4)
		52~68:9~16:13~27 = 4	26 (10)
		50~70:8~17:12~30 = 2	43 (17)
		Otherwise = 0	171 (68)
Fatty acid ratio (PUFA:MUFA:SFA)	0–4 points	P/S = 1~1.5 and M/S = 1~1.5 = 4	54 (22)
		Else if P/S = 0.8~1.7 and M/S = 0.8~1.7 = 2	84 (34)
		Otherwise = 0	112 (45)

RDA: recommended dietary allowance; AI: adequate intakes; MUFA: monounsaturated fatty acids; SFA, saturated fatty acids; PUFA: polyunsaturated fatty acids; P/S, ratio of PUFA to SFA intake; M/S, ratio of MUFA to SFA intake. ^1^ *n* = 250. ^2^ Used as a continuous variable. ^3^ Based on 1700 kcal (7118 kJ)/2200 kcal (9211 kJ)/2700 kcal (11,304 kJ) diet; 1 kcal = 4.1868 kJ. ^4^ Based on sodium from food sources, excluding the discretionary use of table salt. ^5^ Ratio of energy from carbohydrate to protein to fat.

**Table 2:** Characteristics of study participants (*n* = 250) at early pregnancy (<18 weeks’ gestation).

Subject Characteristics	Total Sample ^1^ Median (IQR) or *n* (%)
**Age (years)**	27 (24–32)
**Gestational age (weeks)**	14 (12–16)
**Parity**	
Nulliparous	74 (30)
Primiparous	88 (35)
Multiparous	88 (35)
**Calculated pre-pregnancy BMI (kg/m^2^) ^2^**	25 (22–30)
Underweight (<18.5 kg/m^2^)	14 (6)
Normal weight (18.5–24.99 kg/m^2^)	105 (42)
Overweight (25–29.99 kg/m^2^)	73 (29)
Obese (≥30 kg/m^2^)	57 (23)
**MUAC (cm)**	30 (27–33)
Underweight or chronic wasting (≤23 cm)	10 (4)
Normal weight and overweight (23.1–32.9 cm)	176 (70)
Obese (≥33 cm)	64 (26)
**HIV status**	
Positive	64 (26)
Negative	186 (74)
**Vomiting ^3^**	
Experienced vomiting	111 (48)
Number of days	2 (1–4)
**Nausea** ^3^	
Experienced nausea	122 (52)
Number of days	2 (1–4)
**Ethnicity**	
Black African	219 (88)
Mixed ancestry	28 (11)
Indian	1 (<1)
White	1 (<1)
**Country of birth**	
South Africa	172 (72)
Zimbabwe	60 (25)
Lesotho	4 (2)
Swaziland	3 (1)
**Highest level of education**	
Primary/None	9 (4)
Grade 8–10	37 (15)
Grade 11–12	145 (58)
Tertiary	58 (23)
**Employment**	
Unemployed	123 (49)
Self-employed	11 (4)
Wage-earner	111 (45)
Other	4 (2)
**Marital status**	
Unmarried	100 (40)
Married	68 (27)
Living together	57 (23)
Traditional marriage ^4^	22 (9)
Divorced/Separated	2 (1)
**Social grant(s) received by household**	
None	162 (67)
Child support	69 (29)
Social relief	3 (1)
Disability	2 (1)
Old-age pension	6 (3)
**Living standard measure (LSM)**	
Low (LSM 1–4)	17 (7)
Medium (LSM 5–7)	148 (59)
High (LSM 8–10)	85 (34)
**Household-level food insecurity ^5^**	
No risk for household food insecurity	130 (53)
At risk for household food insecurity	114 (47)
**Risk for prenatal depression ^6^**	5 (2–9)
No/low risk for prenatal depression	183 (74)
At risk for prenatal depression	63 (26)
**Risk for prenatal fatigue ^7^**	19 (1–28)
No/low severity for prenatal fatigue (GFI < 27.9)	179 (74)
More severe level of prenatal fatigue (GFI score ≥ 28)	62 (26)

IQR: interquartile range; BMI: body mass index; MUAC: mid-upper arm circumference; HIV: human immunodeficiency virus; LSM: living standard measure; CCHIP: Community Childhood Hunger Identification Project; EPDS: Edinburgh Postnatal Depression Score; GFI: Global Fatigue Index. Data presented as *n* (%) for categorical variables and median (IQR) for continuous variables. ^1^ Missing data: approximate pre-pregnancy BMI (*n* = 1); vomiting and nausea (*n* = 17); ethnicity (*n* = 1); country of birth (*n* = 11); level of education (*n* = 1); employment (*n* = 1); marital status (*n* = 1); social grants received by household (*n* = 8); household-level food security (*n* = 6); risk for prenatal depression (*n* = 4); risk for prenatal fatigue (*n* = 9). ^2^ Maternal weight was adjusted according to the Institute of Medicine’s guidelines [58] for gestational weight gain to determine an approximate pre-pregnancy BMI. ^3^ Nausea and vomiting experienced within 7 days after enrolment. ^4^ A traditional marriage does not require approval from an officer of validation but is rather entered between parties based on tradition. Recognized under customary law in South Africa, traditional marriage also allows for polygamous marriage. ^5^ Determined using the CCHIP index’s questions on household-level food insecurity. ^6^ Determined using the Edinburgh Postnatal Depression Score [52,53]; a score of 9 or more out of a possible 30 is indicative of a risk for depressive symptoms. ^7^ Determined using the Multidimensional Assessment of Fatigue scale from which the Global Fatigue Index was calculated [51]; a cut-off value of 28 out of a possible 50 was used [54], with a higher score indicating greater severity of fatigue.

**Table 3:** Median (IQR) scores for DQI-I components (*n* = 250).

Category and Component	Score Range (Points)	Median (IQR)
**Total DQI score**	**0–100 points**	**60 (54–66)**
**Variety**	**0–20 points**	**17 (14–20)**
Overall food group variety	0–15 points	12 (12–15)
Within-group variety for protein sources	0–5 points	5 (3–5)
**Adequacy**	**0–40 points**	**31 (28–33)**
Vegetable group	0–5 points	2 (1–3)
Fruit group	0–5 points	4(3–5)
Grain group	0–5 points	4 (3–5)
Fiber	0–5 points	5 (4–5)
Protein	0–5 points	5 (5–5)
Iron	0–5 points	3 (3–4)
Calcium	0–5 points	3 (2–5)
Vitamin C	0–5 points	5 (4–5)
**Moderation**	**0–30 points**	**9 (6–15)**
Total fat	0–6 points	0 (0–3)
Saturated fat	0–6 points	3 (0–3)
Cholesterol	0–6 points	3 (0–6)
Sodium ^1^	0–6 points	0 (0–3)
Empty calories	0–6 points	3 (0–3)
**Overall balance**	**0–10 points**	**2 (0–4)**
Macronutrient ratio	0–6 points	0 (0–2)
Fatty acid ratio	0–4 points	2 (0–2)

IQR: interquartile range; DQI-I: Diet Quality Index-International. ^1^ Based on sodium from food sources, excluding the discretionary use of table salt.

**Table 4:** Nutrient patterns and factor loadings extracted using factor analysis during early pregnancy (*n* = 250).

Nutrients and Variance Explained	Factors (Nutrient Patterns) ^1^		
	Factor 1	Factor 2	Factor 3
	(Plant Protein, Iron, Thiamine, and Folic Acid)	(Animal Protein, Copper, Vitamin A, and Vitamin B_12_)	(Fatty Acids and Sodium)
Animal protein	**−0.46**	**0.53**	0.32
Plant protein	**0.86**	−0.09	−0.00
Saturated fat	**−0.68**	0.08	**0.44**
Monounsaturated fat	**−0.51**	0.08	**0.67**
Polyunsaturated fat	−0.15	−0.08	**0.57**
Cholesterol	−0.09	**0.58**	0.32
Carbohydrates	**0.61**	−0.21	**−0.70**
Total sugars	−0.23	0.05	**−0.68**
Total fiber	**0.70**	0.10	−0.24
Calcium	**−0.44**	0.20	−0.34
Iron	**0.76**	0.35	0.02
Sodium	0.04	0.01	**0.64**
Zinc	**0.45**	**0.44**	0.30
Copper	0.25	**0.79**	−0.08
Vitamin A	0.12	**0.81**	−0.07
Thiamine	**0.77**	0.10	−0.05
Riboflavin	0.01	**0.63**	−0.14
Niacin	**0.54**	0.39	0.31
Vitamin B_6_	**0.67**	0.03	0.26
Folic acid	**0.73**	0.36	−0.07
Vitamin B_12_	0.10	**0.86**	−0.03
Vitamin E % of variance Total variance: 57%	0.04 27%	0.03 18%	**0.41** 12%

^1^ Factor analysis performed on 22 nutrients; estimates presented after rotation. Nutrients considered to have a strong association with the nutrient patterns (factor loading ≤0.4 and ≥0.4) are indicated in bold.

**Table 5:** DQI-I total and category scores by tertiles of nutrient pattern scores (*n* = 250).

DQI-I Categories	Nutrient Pattern 1 Scores				Nutrient Pattern 2 Scores				Nutrient Pattern 3 Scores			
	(Plant Protein, Iron, Thiamine, and Folic Acid)				(Animal Protein, Copper, Vitamin A, and Vitamin B_12_)				(Fatty Acids and Sodium)			
	Tertile 1	Tertile 2	Tertile 3		Tertile 1	Tertile 2	Tertile 3		Tertile 1	Tertile 2	Tertile 3	
	Median (IQR)	Median (IQR)	Median (IQR)	*p*-Value	Median (IQR)	Median (IQR)	Median (IQR)	*p*-Value	Median (IQR)	Median (IQR)	Median (IQR)	*p*-Value
Total score	56.0 ^c^ (52.2–60.4)	60.4 ^b^ (55.3–64.9)	65.2 ^a^ (58.3–71.3)	**<0.001**	60.4 (52.7–65.8)	60.4 (54.7–65.2)	59.5 (54.0–66.2)	0.889	65.8 ^a^ (58.0–73.1)	59.8 ^b^ (54.8–64.1)	56.4 ^c^ (49.9–60.5)	**<0.001**
Variety	17.0 ^a^ (15.0–20.0)	17.0 ^a^ (14.0–20.0)	15.0 ^b^ (10.0–17.0)	**<0.001**	15.0 ^b^ (11.0–18.0)	17.0 ^a^ (14.0–20.0)	17.0 ^a^ (15.0–20.0)	**<0.001**	15.0 ^b^ (10.0–18.0)	17.0 ^a^ (15.0–20.0)	17.0 ^a^ (14.0–20.0)	**<0.001**
Adequacy	30.3 (27.8–33.9)	30.8 (27.6–35.1)	31.4 (29.0–32.7)	0.980	29.8 ^b^ (27.3–32.5)	31.0 ^a,b^ (28.2–33.2)	31.7 ^a^ (29.0–35.1)	**0.040**	30.3 (27.4–32.5)	31.3 (28.4–34.6)	31.8 (28.0–34.2)	0.168
Moderation	6.0 ^c^ (3.0–9.0)	9.0 ^b^ (6.0–15.0)	15.0 ^a^ (9.0–21.0)	**<0.001**	12.0 ^a^ (6.0–18.0)	9.0 ^b^ (6.0–15.0)	9.0 ^b^ (3.0–15.0)	**0.009**	18.0 ^a^ (12.0–24.0)	9.0 ^b^ (6.0–12.0)	6.0 ^c^ (3.0–9.0)	**<0.001**
Overall balance	0.0 ^c^ (0.0–2.0)	2.0 ^b^ (2.0–4.0)	4.0 ^a^ (2.0–6.0)	**<0.001**	2.0 (2.0–4.0)	2.0 (0.0–4.0)	2.0 (0.0–4.0)	**0.045**	4.0 ^a^ (2.0–6.0)	2.0 ^b^ (0.0–4.0)	1.0 ^c^ (0.0–2.0)	**<0.001**

IQR: interquartile range. Significant difference between the median of the tertiles indicated in bold (*p* < 0.05) (according to Welsh test). Medians with a different letter in their superscript are significantly different at the 0.05 level according to the Games–Howell test.

**Table 6:** Associations of maternal and household factors with nutrient patterns and DQI-I ^1^.

	Nutrient Pattern 1 Plant Protein, Iron, Thiamine, and Folic Acid			Nutrient Pattern 2 Animal Protein, Copper, Vitamin A, and Vitamin B_12_			Nutrient Pattern 3 Fatty Acids and Sodium			DQI-I		
	β	SE	*p*-Value	β	SE	*p*-Value	β	SE	*p*-Value	β	SE	*p*-Value
**Step 1** ^2^												
**Maternal age**												
<27 years vs. ≥27 years (RC)	−2.5	0.8	**0.002**	0.0	0.5	1.000	0.8	0.4	0.060	−5.0	1.3	**<0.001**
**Education**												
≤Grade 10	2.4	1.0	**0.012**	−0.4	0.6	0.496	−0.8	0.5	0.128	−0.7	1.6	0.678
Grade 11–12 (RC)												
Tertiary	−0.9	0.8	0.291	−0.2	0.5	0.695	0.6	0.5	0.168	−1.3	1.4	0.359
**Marital status**												
Married/cohabitating vs. Single (RC)	0.7	0.7	0.321	−1.0	0.5	0.042	−0.6	0.4	0.157	−0.5	1.2	0.699
**Step 2** ^2^												
**Maternal age**												
<27 years vs. ≥27 years (RC)	−2.1	0.8	**0.009**	−0.2	0.5	0.749	0.7	0.4	0.103	−4.8	1.4	**0.001**
**Education**												
≤Grade 10	1.9	1.0	**0.051**	−0.3	0.6	0.593	−0.8	0.5	0.149	−0.2	1.6	0.483
Grade 11–12 (RC)												
Tertiary	−0.4	0.9	0.687	−0.4	0.6	0.518	0.6	0.5	0.186	−0.8	1.5	0.616
**Marital status**												
Married/cohabitating vs. Single (RC)	0.7	0.8	0.336	−0.9	0.5	0.067	−0.6	0.4	0.154	−0.5	1.3	0.678
**Employment**												
Unemployed vs. Employed (RC)	−0.8	0.7	0.256	0.6	0.5	0.209	0.4	0.4	0.290	−0.5	1.2	0.707
**Grants received by the household**												
Not received vs. Received (RC)	−0.9	0.8	0.279	−0.2	0.5	0.728	0.0	0.4	0.934	−1.0	1.4	0.451
	**β**	**SE**	***p*-Value**	**β**	**SE**	***p*-Value**	**β**	**SE**	***p*-Value**	**β**	**SE**	***p*-Value**
**Household-level food insecurity**												
No risk vs. At risk (RC)	−0.8	0.7	0.282	−0.1	0.5	0.823	−0.2	0.4	0.687	−1.8	1.3	0.170
**Living standard measure**												
Low living standard measure (1–5) vs. High living standard measure (6–10) (RC)	2.1	1.0	**0.030**	−0.9	0.6	0.159	−0.3	0.5	0.618	0.8	1.7	0.639
**Step 3** ^2^												
**Maternal age**												
<27 years vs. ≥27 years (RC)	−1.9	0.9	**0.046**	0.1	0.6	0.918	0.6	0.5	0.249	−5.8	1.6	**0.001**
**Education**												
≤Grade 10	2.0	1.0	**0.034**	−0.3	0.6	0.620	−0.8	0.5	0.129	−0.9	1.7	0.593
Grade 11–12 (RC)												
Tertiary	−0.1	0.9	0.889	−0.2	0.6	0.701	0.6	0.5	0.257	−1.1	1.6	0.501
**Marital status**												
Married/cohabitating vs. Single (RC)	1.2	0.8	0.128	−0.7	0.5	0.161	−0.7	0.4	0.084	−0.5	1.3	0.699
**Employment**												
Unemployed vs. Employed (RC)	−0.8	0.7	0.278	0.5	0.5	0.262	0.4	0.4	0.316	−0.1	1.3	0.920
**Grants received by the household**												
Not received vs. Received (RC)	−1.0	0.8	0.219	−0.3	0.5	0.587	0.1	0.4	0.849	−0.7	1.4	0.611
**Household-level food insecurity**												
No risk vs. At risk (RC)	−0.8	0.7	0.278	−0.1	0.5	0.880	−0.2	0.4	0.710	−1.9	1.3	0.139
**Living standard measure**												
Low living standard measure (1–5) vs.	2.0	1.0	**0.049**	−1.1	0.6	0.101	−0.3	0.5	0.593	1.0	1.7	0.572
High living standard measure (6–10) (RC)												
**Parity**												
Nulliparous vs. Primi- and multiparous (RC)	−1.1	1.0	0.253	−0.6	0.6	0.341	0.5	0.5	0.385	1.3	1.7	0.455
**Nausea experienced** ^3^												
Yes vs. No (RC)	−1.0	0.7	0.185	−0.3	0.5	0.591	0.3	0.4	0.414	−0.7	1.3	0.558
**Vomiting experienced** ^3^												
Yes vs. No (RC)	0.6	0.7	0.793	0.5	0.5	0.263	0.1	0.4	0.765	−0.2	1.3	0.862
**HIV status**												
Negative vs. Positive (RC)	1.6	0.8	0.055	0.3	0.5	0.602	−0.6	0.5	0.205	1.9	1.4	0.180
**Step 4** ^2^												
**Maternal age**												
<27 years vs. ≥27 years (RC)	−1.9	0.9	0.050	0.1	0.6	0.862	0.6	0.5	0.253	−5.7	1.6	0.001
**Education**												
≤Grade 10	2.1	1.0	**0.032**	−0.3	0.6	0.685	−0.8	0.5	0.131	−0.8	1.7	0.612
Grade 11–12 (RC)												
Tertiary	−0.1	0.9	0.914	−0.2	0.6	0.744	0.6	0.5	0.262	−1.0	1.6	0.513
**Marital status**												
Married/cohabitating vs. Single (RC)	1.2	0.8	0.120	−0.7	0.5	0.153	−0.7	0.4	0.083	−0.5	1.4	0.699
**Employment**												
Unemployed vs. Employed (RC)	−0.8	0.7	0.297	0.5	0.5	0.269	0.4	0.4	0.331	−0.1	1.3	0.914
	**β**	**SE**	***p*-Value**	**β**	**SE**	***p*-Value**	**β**	**SE**	***p*-Value**	**β**	**SE**	***p*-Value**
**Grants received by the household**												
Not received vs. Received (RC)	−1.0	0.8	0.206	−0.3	0.5	0.501	0.1	0.4	0.849	−0.8	1.4	0.590
**Household-level food insecurity**												
No risk vs. At risk (RC)	−0.8	0.7	0.292	0.0	0.5	0.953	−0.2	0.4	0.712	−1.9	1.3	0.147
**Living standard measure**												
Low living standard measure (1–5) vs.	2.0	1.0	**0.049**	−1.2	0.6	0.068	−0.3	0.5	0.577	0.9	1.7	0.605
High living standard measure (6–10) (RC)												
**Parity**												
Nulliparous vs. Primi- and multiparous (RC)	−1.1	1.0	0.238	−0.6	0.6	0.314	0.5	0.5	0.379	1.2	1.7	0.464
**Nausea experienced** ^3^												
Yes vs. No (RC)	−0.9	0.7	0.239	−0.1	0.5	0.789	0.3	0.4	0.436	−0.6	1.3	0.612
**Vomiting experienced** ^3^												
Yes vs. No (RC)	0.6	0.7	0.454	0.5	0.5	0.258	0.1	0.4	0.751	−0.2	1.3	0.862
**HIV status**												
Negative vs. Positive (RC)	1.5	0.8	0.073	0.3	0.5	0.617	−0.6	0.5	0.230	1.9	1.5	0.188
**Risk for prenatal depression**												
At risk vs. No risk (RC)	0.8	1.5	0.598	2.6	1.0	0.008	0.1	0.8	0.891	1.8	2.6	0.485
**Risk for prenatal fatigue**												
Global Fatigue Index (continues)	0.6	0.8	0.445	0.1	0.5	0.873	−0.2	0.4	0.716	0.1	1.4	0.961

β: unstandardized coefficients; SE: standard errors; RC: reference category after categorical coding in regression analysis (dummy variables); HIV: human immunodeficiency virus. ^1^ Grey sections indicates regression coefficients of models which did not have a significant model fit. ^2^ Step 1: sociodemographic factors; step 2: sociodemographic and socioeconomic factors; step 3: sociodemographic-, socioeconomic-, and pregnancy-related factors and HIV status; step 4: sociodemographic-, socioeconomic-, and pregnancy-related factors, HIV status and psychological factors. ^3^ Nausea and vomiting experienced within 7 days after enrolment. Significance (*p* < 0.05) indicated in bold. Background colour indicates that these models did not have a significant model fit during the statistical analysis.

## Data Availability

As required by the Human Research Ethics Committee of the North-West University, South Africa, the researcher signed confidentiality agreements in which they agreed “not to disclose confidential information to anyone other than North West University unless required to do so by law or by court order”. Participant consent forms were also clear for the purposes of data usage. Permission to obtain the data can be obtained from the North-West University Health Research Ethics Committee, Wayne Towers: wayne.towers@nwu.ac.za.

## Supplementary Materials

The following are available online at https://www.mdpi.com/2072-6643/13/2/565/s1, Table S1: Consumption of food groups as a percentage of total energy intake (median and interquartile range) according to nutrient pattern 1 (*n* = 250), Table S2: Consumption of food groups as a percentage of total energy intake (median and interquartile range) according to nutrient pattern 2 (*n* = 250), Table S3: Consumption of food groups as a percentage of total energy intake (median and interquartile range) according to nutrient pattern 3 (*n* = 250).
