# Supplementary material for: A Priori and a Posteriori Dietary Patterns among Pregnant Women in Johannesburg, South Africa: The NuPED Study
Source: Nutrients. 2021 Feb 9;13(2):565. doi: 10.3390/nu13020565 (PMC7914963; doi:10.3390/nu13020565)
Supplement: Supplementary file 1 [file nutrients-13-00565-s001.pdf]

Table S1: Consumption of food groups as a percentage of total energy intake (median and interquartile range) according nutrient pattern 1 (n = 250)

	Nutrient pattern 1 *Plant protein, iron, thiamine and folic acid*						
	Tertile 1		Tertile 2		Tertile 3		
	**Median**	**IQR**	**Median**	**IQR**	**Median**	**IQR**	**p-value**
Breakfast cereals	1.44	(0.00 – 3.00)	0.82	(0.00 – 3.21)	0.72	(0.00 – 3.67)	0.203
Cakes, biscuits and pudding	3.62[a]	(0.97 – 7.37)	3.14[a]	(0.60 – 6.22)	1.23[b]	(0.00 – 3.21)	**<0.001**
Cheese	0.50[a]	(0.00 -1.51)	0.13[a,b]	(0.00 – 0.81)	0.00[b]	(0.00 – 0.52)	**0.021**
Chicken	3.41	(1.55 – 6.21)	3.37	(1.68 – 5.40)	2.37	(0.83 – 5.42)	0.411
Crackers	0.00	(0.00 – 0.00)	0.00	(0.00 – 0.00)	0.00	(0.00 – 0.00)	0.423
Dairy[1]	5.33[a]	(3.12 – 7.19)	5.15[a,b]	(2.24 – 7.99)	2.87[b]	(1.11 – 6.30)	**0.019**
Decaffeinated tea	0.00	(0.00 – 0.04)	0.01	(0.00 – 0.05)	0.01	(0.00 – 0.06)	0.170
Eggs	2.14	(0.47 – 3.76)	1.77	(0.00 – 3.99)	1.38	(0.34 – 4.31)	0.825
Fatty foods[2]	4.47[a]	(2.07 – 7.27)	2.93[a,b]	(1.33 – 5.68)	1.99[b]	(0.00 – 5.81)	**0.005**
Fish and seafood	1.08	(0.28 – 2.57)	0.87	(0.28 – 2.01)	0.62	(0.06 – 1.84)	0.204
Fresh and dried fruit	7.35	(4.62 – 10.84)	7.28	(4.76 – 9.60)	8.61	(5.10 – 11.68)	0.282
Fruit juice	2.38[a]	(0.68 – 4.36)	0.79[a,b]	(0.00 – 3.02)	0.29[b]	(0.00 – 2.38)	**0.001**
Leafy vegetables	0.34	(0.08 – 1.08)	073	(0.21 – 1.96)	0.85	(0.27 – 1.90)	0.184
Legumes	1.01[b]	(0.06 – 1.95)	1.01[a,b]	(0.04 – 2.23)	1.18[a]	(0.00 – 3.20)	**0.011**
Maize	4.68[c]	(1.88 – 8.12)	11.03[b]	(5.42 – 15.53)	15.23[a]	(8.34 – 22.63)	**<0.001**
Margarine	0.28	(0.00 – 0.80)	0.14	(0.00 – 0.94)	0.00	(0.00 – 0.87)	0.144
Miscellaneous[3]	0.61	(0.11 – 1.77)	0.53	(0.07 – 1.41)	0.30	(0.00 – 1.24)	0.379
Nuts and nut spreads	0.53	(0.00 – 1.39)	0.42	(0.00 – 1.85)	0.67	(0.00 – 3.12)	0.373
Organ meat and offal	0.41	(0.00 – 1.45)	0.70	(0.00 – 2.39)	0.60	(0.00 – 2.20)	0.062
Cooked porridge[4]	0.25	(0.00 – 1.80)	0.53	(0.00 – 3.84)	0.35	(0.00 – 3.96)	0.454
Other vegetables[5]	0.99	(0.48 – 1.69)	1.07	(0.33 – 2.62)	1.30	(0.46 – 3.00)	**0.043**
Potatoes and sweet potatoes	0.88	(0.34 – 2.34)	1.30	(0.34 – 2.54)	0.83	(0.17 – 2.98)	0.416
Processed meat	4.16[a]	(1.74 – 6.69)	2.36[b,c]	(0.77 – 4.23)	1.62[c]	(0.14 – 4.20)	**<0.001**
Red meat	5.75[a]	(2.39 – 10.09)	2.74[b]	(0.32 – 6.52)	1.42[c]	(0.10 – 3.74)	**<0.000**
Relish	0.15	(0.00 – 0.73)	0.35	(0.00 – 1.47)	0.00	(0.00 – 0.62)	0.065
Rice and Pasta	4.56	(2.54 – 7.30)	3.95	(2.21 – 6.26)	2.70	(1.10 – 5.41)	**<0.049**
Roast potatoes and chips	1.61	(0.25 – 5.06)	1.50	(0.00 – 4.70)	1.34	(0.00 – 4.19)	0.655
Salad oils	0.86[a]	(0.08 – 3.29)	0.58[a,b]	(0.00 – 1.88)	0.02[b]	(0.00 – 1.23)	**0.001**
Salad vegetables	0.33[a,b]	(0.13 – 0.63)	0.34[a]	(0.05 – 0.72)	0.22[b]	(0.00 – 0.40)	**0.026**
Savoury snacks	3.10	(0.87 – 6.67)	2.17	(0.75 – 5.51)	2.37	(0.56 – 6.19)	0.276
Sugar	2.40	(0.59 – 4.01)	2.13	(1.05 – 4.23)	2.22	(0.61 – 4.68)	0.591
Sugar-sweetened drinks	1.63[a]	(0.33 – 3.20)	1.81[a]	(0.75 – 3.10)	0.67[b]	(0.00 – 2.07)	**0.015**
Sugar-sweetened dairy[6]	0.98[a]	(0.00 – 2.89)	0.62[a]	(0.00 – 2.17)	0.00[b]	(0.00 – 0.83)	**<0.001**
Sweet spreads and jam	0.00	(0.00 – 0.41)	0.00	(0.00 – 0.00)	0.00	(0.00 – 0.00)	0.120
Sweets and chocolates	1.59[a]	(0.00 – 5.07)	0.54[b,c]	(0.00 – 1.49)	0.00[c]	(0.00 – 1.68)	**0.002**
Tea and coffee	0.01	(0.00 – 0.08)	0.00	(0.00 – 0.08)	0.00	(0.00 – 0.07)	0.470
Vitamin A rich vegetables	0.50	(0.19 – 0.96)	0.42	(0.09 – 0.85)	0.34	(0.00 – 0.76)	0.374
White bread	2.39	(0.00 – 4.67)	1.10	(0.00 – 6.54)	0.00	(0.00 – 6.18)	0.502
Whole wheat and brown bread	0.00[c]	(0.00 – 2.60)	2.51[b]	(0.00 – 6.18)	4.86[a]	(0.00 – 12.86)	**<0.001**

IQR: interquartile range

[1]Including both full fat and skim dairy products.

[2]Pies, 'vetkoek', pizza, hamburgers and pizza.

[3]Soup powders, sauces (chutney, tomato, mushroom and white), and meat and vegetable extract bread spreads.
[4]Oats and sorghum porridges.
[5]Including amongst others beetroot, broccoli, cauliflower, mealies, tomatoes, cabbage, green beans and sweet peppers.
[6]Ice cream, milk shake and malted milk beverages.
Significant difference between the median of the tertiles indicated in **bold** ($p<0.05$) (according to Welsh test)
Medians with a different letter in their superscript are significantly different at the 0.05 level according to a Games-Howell test.

Table S2: Consumption of food groups as a percentage of total energy intake (median and interquartile range) according nutrient pattern 2 (n = 250)

	Nutrient pattern 2 *Animal protein, copper, vitamin A and vitamin B_{12}*						
	Tertile 1		Tertile 2		Tertile 3		
	Median	IQR	Median	IQR	Median	IQR	p-value
Breakfast cereals	0.74	(0.00 - 2.79)	1.74	(0.00 - 3.78)	0.72	(0.00 - 3.27)	0.105
Cakes, biscuits and pudding	2.87	(0.67 - 7.13)	2.92	(0.73 - 5.61)	1.56	(0.00 - 4.29)	0.183
Cheese	0.12[b]	(0.00 - 0.66)	0.21[a,b]	(0.00 - 0.72)	0.36[a]	(0.00 - 1.57)	**0.022**
Chicken	2.18[c]	(0.82 - 4.70)	3.83[a,b]	(2.10 - 5.52)	2.86[b]	(1.46 - 6.85)	**0.006**
Crackers	0.00	(0.00 - 0.00)	0.00	(0.00 - 0.00)	0.00	(0.00 - 0.00)	0.974
Dairy[1]	3.90	(2.35 - 6.79)	4.97	(2.27 - 7.86)	4.71	(1.73 - 7.47)	0.504
Decaffeinated tea	0.01	(0.00 - 0.05)	0.01	(0.00 - 0.05)	0.01	(0.00 - 0.06)	0.317
Eggs	1.09[b]	(0.00 - 2.72)	1.97[b,c]	(0.31 - 3.75)	3.16[a]	(0.96 - 5.53)	**<0.001**
Fatty foods[2]	4.47[a]	(1.25 - 7.66)	2.77[a,b]	(1.15 - 6.10)	2.40[b]	(0.78 - 4.85)	**0.009**
Fish and seafood	0.72[b]	(0.16 - 1.64)	1.10[a,b]	(0.20 - 2.30)	0.80[a]	(0.28 - 2.63)	**0.036**
Fresh and dried fruit	7.09	(4.76 - 10.04)	7.62	(4.61 - 10.31)	8.35	(5.42 - 11.40)	0.251
Fruit juice	1.17	(0.00 - 4.29)	1.20	(0.00 -3.19)	0.91	(0.00 - 2.74)	0.265
Leafy vegetables	0.48	(0.11 - 1.15)	0.65	(0.18 - 1.63)	0.75	(0.29 - 2.00)	0.155
Legumes	1.31	(0.15 - 2.20)	0.84	(0.00 - 2.75)	0.98	(0.00 - 2.23)	0.836
Maize	12.44	(4.38 - 19.77)	9.37	(4.60 - 14.71)	8.12	(3.94 - 14.16)	0.066
Margarine	0.21	(0.00 - 0.96)	0.33	(0.00 - 0.84)	0.00	(0.00 - 0.66)	0.070
Miscellaneous[3]	0.36	(0.08 - 1.10)	0.65	(0.09 - 1.85)	0.49	(0.00 - 1.27)	0.294
Nuts and nut spreads	0.63	(0.00 - 1.82)	0.43	(0.00 - 2.76)	0.69	(0.00 - 3.07)	**0.044**
Organ meat and offal	0.18[b]	(0.00 - 0.69)	0.44[b,c]	(0.00 - 1.26)	1.53[a]	(0.36 - 3.49)	**<0.001**
Cooked porridge[4]	0.06	(0.00 - 3.38)	0.56	(0.00 - 2.24)	0.60	(0.00 - 2.93)	0.432
Other vegetables[5]	0.68[b]	(0.20 - 1.76)	1.10[a,b]	(0.50 - 2.63)	1.30[a]	(0.55 - 2.53)	**0.030**
Potatoes and sweet potatoes	1.10	(0.24 - 2.28)	0.85	(0.25 - 2.33)	1.31	(0.25 - 2.71)	0.694
Processed meat	2.22	(0.79 - 5.30)	2.99	(1.31 - 4.75)	2.73	(0.59 - 4.92)	0.584
Red meat	1.58	(0.00 - 5.75)	3.75	(0.78 - 6.90)	3.38	(1.30 - 7.30)	0.087
Relish	0.12	(0.00 - 1.06)	0.20	(0.00 - 0.87)	0.12	(0.00 - 0.95)	0.460
Rice and Pasta	3.15	(1.54 - 6.13)	3.91	(2.08 - 6.23)	4.18	(2.01 - 6.73)	0.922
Roast potatoes and chips	3.01[a]	(0.19 - 6.88)	1.84[b,c]	(0.00 - 4.23)	0.51[c]	(0.00 - 2.52)	**0.002**
Salad oils	0.43	(0.00 - 1.73)	0.67	(0.00 - 1.97)	0.22	(0.00 - 2.22)	0.632
Salad vegetables	0.28	(0.03 - 0.48)	0.28	(0.08 - 0.56)	0.31	(0.05 - 0.69)	0.311
Savoury snacks	2.38	(0.85 - 6.15)	3.34	(1.07 - 6.80)	1.86	(0.22 - 5.37)	0.795
Sugar	2.99[a]	(1.41 - 4.87)	2.67[a]	(1.07 - 4.80)	1.61[b]	(0.44 - 3.21)	**<0.001**
Sugar-sweetened drinks	1.16	(0.13 - 3.04)	1.67	(0.44 - 3.09)	1.11	(0.15 - 2.20)	0.124
Sugar-sweetened dairy[6]	0.59	(0.00 - 1.74)	0.67	(0.00 - 1.97)	0.43	(0.00 - 1.46)	0.733
Sweet spreads and jam	0.00	(0.00 - 0.19)	0.00	(0.00 - 0.06)	0.00	(0.00 - 0.00)	0.204
Sweets and chocolates	0.55	(0.00 - 3.16)	0.54	(0.00 - 2.53)	0.33	(0.00 - 2.16)	0.939
Tea and coffee	0.01	(0.00 - 0.09)	0.00	(0.00 - 0.08)	0.00	(0.00 - 0.06)	0.244
Vitamin A rich vegetables	0.38	(0.04 - 0.75)	0.34	(0.00 - 0.80)	0.53	(0.18 - 1.28)	0.052
White bread	2.60[a]	(0.00 - 8.81)	0.71[a,b]	(0.00 - 5.87)	0.34[b]	(0.00 - 4.13)	**0.032**
Whole wheat and brown bread	0.00	(0.00 - 5.43)	3.19	(0.00 - 7.42)	2.01	(0.00 - 7.53)	0.320

IQR: interquartile range

[1]Including both full fat and skim dairy products.

[2]Pies, 'vetkoek', pizza, hamburgers and pizza.

[3]Soup powders, sauces (chutney, tomato, mushroom and white), and meat and vegetable extract bread spreads.
[4]Oats and sorghum porridges.
[5]Including amongst others beetroot, broccoli, cauliflower, mealies, tomatoes, cabbage, green beans and sweet peppers.
[6]Ice cream, milk shake and malted milk beverages.
Significant difference between the median of the tertiles indicated in **bold** ($p<0.05$) (according to Welsh test)
Medians with a different letter in their superscript are significantly different at the 0.05 level according to a Games-Howell test.

Table S3: Consumption of food groups as a percentage of total energy intake (median and interquartile range) according nutrient pattern 3 (n = 250)

	**Nutrient pattern 3** *Fatty acids and sodium*						
	**Tertile 1**		**Tertile 2**		**Tertile 3**		
	**Median**	**IQR**	**Median**	**IQR**	**Median**	**IQR**	**p-value**
Breakfast cereals	0.72	(0.00 - 4.10)	0.85	(0.00 - 2.55)	1.73	(0.00 - 3.31)	0.081
Cakes, biscuits and pudding	1.57	(0.09 - 4.35)	3.10	(1.21 - 5.99)	2.87	(0.17 - 6.75)	0.078
Cheese	0.00[b]	(0.00 - 0.41)	0.18[a,b]	(0.00 - 0.76)	0.50[a]	(0.00 - 1.69)	**0.014**
Chicken	2.16	(0.81 - 5.39)	3.37	(1.78 - 6.54)	3.37	(1.81 - 5.62)	0.167
Crackers	0.00	(0.00 - 0.00)	0.00	(0.00 - 0.00)	0.00	(0.00 - 0.00)	0.680
Dairy[1]	5.86[a]	(218 - 10.43)	4.37[b,c]	(2.27 - 7.13)	3.39[c]	(1.98 - 6.39)	**0.006**
Decaffeinated tea	0.01	(0.00 - 0.06)	0.01	(0.00 - 0.06)	0.00	(0.00 - 0.03)	0.085
Eggs	0.65	(0.00 - 3.25)	2.14	(0.74 - 4.24)	2.34	(0.80 - 3.88)	0.135
Fatty foods[2]	1.57[c]	(0.31 - 4.00)	3.12[b]	(1.62 - 6.19)	4.47[a]	(2.34 - 8.20)	**<0.001**
Fish and seafood	0.64	(0.07 - 1.96)	1.07	(0.22 - 2.45)	0.92	(0.24 - 2.22)	0.285
Fresh and dried fruit	8.89[a]	(6.12 - 12.34)	7.07[a,b]	(4.59 - 10.83)	6.65[b]	(3.68 - 9.33)	**0.001**
Fruit juice	0.85	(0.00 - 3.26)	1.51	(0.30 - 3.63)	0.86	(0.00 - 3.16)	0.439
Leafy vegetables	0.85	(0.20 - 1.63)	0.49	(0.12 - 1.48)	0.62	(0.14 - 2.19)	0.208
Legumes	1.29	(0.00 - 3.20)	0.73	(0.00 - 1.89)	0.78	(0.06 - 2.19)	0.104
Maize	14.16[a]	(6.89 - 22.58)	10.22[b]	(5.13 - 15.67)	6.01[c]	(1.99 - 10.54)	**<0.001**
Margarine	0.14	(0.00 - 0.62)	0.13	(0.00 - 0.96)	0.30	(0.00 - 0.87)	0.691
Miscellaneous[3]	0.28	(0.00 - 0.73)	0.58	(0.07 - 1.60)	0.77	(0.22 - 1.70)	0.229
Nuts and nut spreads	0.20[b]	(0.00 - 1.32)	0.69[a,b]	(0.00 - 2.18)	0.85[a]	(0.00 - 3.62)	**0.026**
Organ meat and offal	0.24	(0.00 - 1.27)	1.01	(0.00 - 2.48)	0.54	(0.00 - 2.11)	0.259
Cooked porridge[4]	0.68[a]	(0.00 - 5.81)	0.37[a,b]	(0.00 - 2.52)	0.14[b]	(0.00 - 1.80)	**0.004**
Other vegetables[5]	1.10	(0.44 - 2.89)	0.99	(0.34 - 2.06)	1.27	(0.38 - 2.30)	0.867
Potatoes and sweet potatoes	0.88	(0.20 - 2.03)	0.84	(0.22 - 2.34)	1.29	(0.54 - 3.06)	0.262
Processed meat	1.29[c]	(0.12 - 3.03)	2.92[b]	(1.37 - 4.89)	4.28[a]	(1.70 - 7.19)	**<0.001**
Red meat	1.01[c]	(0.00 - 3.84)	2.88[a,b]	(0.67 - 6.65)	4.95[a]	(2.34 - 9.10)	**<0.001**
Relish	0.10	(0.00 - 0.90)	0.08	(0.00 - 1.11)	0.18	(0.00 - 0.95)	0.997
Rice and Pasta	3.66	(1.28 - 6.67)	4.01	(2.22 - 6.24)	3.83	(2.16 - 5.86)	0.835
Roast potatoes and chips	1.14	(0.00 - 4.99)	2.09	(0.35 - 4.45)	1.26	(0.00 - 4.29)	0.613
Salad oils	0.02[c]	(0.00 - 0.84)	0.71[a,b]	(0.00 - 2.30)	0.90[a]	(0.00 - 2.98)	**<0.001**
Salad vegetables	0.15	(0.00 - 0.44)	0.31	(0.09 - 0.63)	0.38	(0.13 - 0.63)	0.208
Savoury snacks	1.61	(0.47 - 5.63)	2.80	(0.66 - 5.27)	3.10	(0.99 - 7.91)	0.219
Sugar	2.86[a]	(0.71 - 6.07)	2.38[b]	(1.11 - 4.16)	1.82[c]	(0.49 - 3.27)	**<0.001**
Sugar-sweetened drinks	0.96[a,b]	(0.00 - 3.04)	1.81[a]	(0.64 - 3.24)	1.11[b]	(0.23 - 2.39)	**0.036**
Sugar-sweetened dairy[6]	0.00	(0.00 - 1.06)	0.74	(0.00 - 1.85)	0.71	(0.00 - 1.98)	0.089
Sweet spreads and jam	0.00	(0.00 - 0.00)	0.00	(0.00 - 0.18)	0.00	(0.00 - 0.07)	0.613
Sweets and chocolates	0.10	(0.00 - 1.70)	0.74	(0.00 - 2.27)	0.64	(0.00 - 3.66)	0.164
Tea and coffee	0.00	(0.00 - 0.04)	0.00	(0.00 - 0.09)	0.03	(0.00 - 0.09)	0.790
Vitamin A rich vegetables	0.34	(0.00 - 0.86)	0.41	(0.17 - 0.87)	0.46	(0.13 - 0.92)	0.971
White bread	0.50	(0.00 - 4.04)	1.78	(0.00 - 7.77)	1.42	(0.00 - 5.93)	0.125
Whole wheat and brown bread	2.00	(0.00 - 7.54)	1.15	(0.00 - 8.00)	2.35	(0.00 - 5.48)	0.485

IQR: interquartile range

[1]Including both full fat and skim dairy products.

[2]Pies, 'vetkoek', pizza, hamburgers and pizza.

[3]Soup powders, sauces (chutney, tomato, mushroom and white), and meat and vegetable extract bread spreads.
[4]Oats and sorghum porridges.
[5]Including amongst others beetroot, broccoli, cauliflower, mealies, tomatoes, cabbage, green beans and sweet peppers.
[6]Ice cream, milk shake and malted milk beverages.
Significant difference between the median of the tertiles indicated in **bold** ($p<0.05$) (according to Welsh test)
Medians with a different letter in their superscript are significantly different at the 0.05 level according to a Games-Howell test.
